# Positive Association between Aspirin-Intolerant Asthma and Genetic Polymorphisms of *FSIP1*: a Case-Case Study

**DOI:** 10.1186/1471-2466-10-34

**Published:** 2010-06-01

**Authors:** Jason Yongha Kim, Jeong Hyun Kim, Tae Joon Park, Joon Seol Bae, Jin Sol Lee, Charisse Flerida Pasaje, Byung Lae Park, Hyun Sub Cheong, Jong-Sook Park, Sung-Woo Park, Soo-Taek Uh, Mi-Kyeong Kim, Inseon S Choi, Sang Heon Cho, Byoung Whui Choi, Choon-Sik Park, Hyoung Doo Shin

**Affiliations:** 1 Department of Life Science, Sogang University, Seoul, Republic of Korea; 2 Department of Genetic Epidemiology, SNP Genetics, Inc., Seoul, Republic of Korea; 3 Genome Research Center for Allergy and Respiratory Disease, Soonchunhyang University Bucheon Hospital, Bucheon, Republic of Korea; 4 Division of Allergy and Respiratory Medicine, Soonchunhyang University Seoul Hospital, Seoul, Republic of Korea; 5 Division of Internal Medicine, Chungbuk National University, College of Medicine, Cheongju, Republic of Korea; 6 Department of Allergy, Chonnam National University Medical School and Research Institute of Medical Sciences, Gwangju, Republic of Korea; 7 Department of Internal Medicine and Institute of Allergy and Clinical Immunology, Seoul National University College of Medicine, Seoul, Republic of Korea; 8 Division of Pulmonology and Allergy, Department of Internal Medicine, Chung-Ang University Yongsan Hospital, Seoul, Republic of Korea

## Abstract

**Background:**

Aspirin-intolerant asthma (AIA), which is caused by non-steroidal anti-inflammatory drugs (NSAIDs) such as aspirin, causes lung inflammation and reversal bronchi reduction, leading to difficulty in breathing. Aspirin is known to affect various parts inside human body, ranging from lung to spermatogenesis. *FSIP1*, also known as *HDS10*, is a recently discovered gene that encodes fibrous sheath interacting protein 1, and is regulated by amyloid beta precursor protein (APP). Recently, it has been reported that a peptide derived from APP is cleaved by α disintegrin and metalloproteinase 33 (*ADAM33*), which is an asthma susceptibility gene. It has also been known that the *FSIP1 *gene is expressed in airway epithelium.

**Objectives:**

Aim of this study is to find out whether *FSIP1 *polymorphisms affect the onset of AIA in Korean population, since it is known that AIA is genetically affected by various genes.

**Methods:**

We conducted association study between 66 single nucleotide polymorphisms (SNPs) of the *FSIP1 *gene and AIA in total of 592 Korean subjects including 163 AIA and 429 aspirin-tolerant asthma (ATA) patients. Associations between polymorphisms of *FSIP1 *and AIA were analyzed with sex, smoking status, atopy, and body mass index (BMI) as covariates.

**Results:**

Initially, 18 SNPs and 4 haplotypes showed associations with AIA. However, after correcting the data for multiple testing, only one SNP showed an association with AIA (corrected *P*-value = 0.03, OR = 1.63, 95% CI = 1.23-2.16), showing increased susceptibility to AIA compared with that of ATA cases. Our findings suggest that *FSIP1 *gene might be a susceptibility gene for aspirin intolerance in asthmatics.

**Conclusion:**

Although our findings did not suggest that SNPs of *FSIP1 *had an effect on the reversibility of lung function abnormalities in AIA patients, they did show significant evidence of association between the variants in *FSIP1 *and AIA occurrence among asthmatics in a Korean population.

## Background

Asthma is a disease that affects a large number of people globally, which is estimated to be about 300 million worldwide [1] and about 3 million asthma patients in South Korea. The disease is defined by the lung inflammation and difficulty of breathing when the suspect is under the influence of various factors that trigger the asthma reaction. Among these factors, non-steroidal anti-inflammatory drugs such as aspirin are known to cause aspirin-intolerant asthma. The AIA was first described in 1922 [2,3], and its most noticeable symptoms include aspirin sensitivity, bronchial asthma, and chronic rhinosinusitis with nasal polyposis [4-6]. When non-steroidal anti-inflammatory drugs such as aspirin are ingested, the drugs cause bronchoconstriction in the patients. Aspirin, also known as acetylsalicylic acid, is primarily used as pain and fever reliever, and as an anti-inflammatory medication. One function of aspirin is decrease of prostaglandin production, with the most significant effect of decrease in the prostaglandin on the alleviation of inflammation and pain.

Recently, there have been several reports that found the relation between AIA and genetic polymorphisms, such as Adenosine A1 receptor (*ADORA1*) polymorphisms 1405C > T and A102A [5]; several polymorphisms in prostaglandin receptors and thromboxane receptor [7]; Interleukin 10 (*IL10*) polymorphism -1082 A > G [8]; cysteinyl leukotriene receptor 1 (*CYSLTR1*) promoter polymorphism -634C > T [6]; angiotensin I converting enzyme (*ACE*) polymorphisms -262 A > T and -115 T > C [9]; and Fc Fragment of IgE (*FCER1G*) polymorphism -237A > G [10]. These results suggest that other genes or complex signal pathways might be related to the development of aspirin hypersensitivity in asthmatics.

Fibrous sheath interacting protein 1 gene (*FSIP1*), also known as *HSD10*, is a recently discovered gene that was first described in 2003 [11]. With its primary function on protein binding, the *FSIP1 *gene is expressed in airway epithelium (GSE4498 and GDS2486, GEO database) [12]. It has been reported that *FSIP1 *is regulated by amyloid beta (Aβ) precursor protein (APP) [13]. APP is an integral membrane protein expressed in many tissues, and particularly, in the synapses of neurons. There has been a report that APP is cleaved by α disintegrin and metalloproteinase 33 *(ADAM33)*, which is an asthma susceptibility gene [14,15]. Based on these findings, we hypothesized *FSIP1 *gene could have an effect on the mechanism of aspirin on the various levels, including onset of AIA, and conducted association analyses of *FSIP1 *gene polymorphisms between AIA and ATA patients. We also conducted association analyses between SNPs of *FSIP1 *and fall rate of FEV_1 _by aspirin provocation.

## Methods

### Study Subjects

This study was conducted in compliance with the Global Initiative for Asthma (GINA) Global strategy for asthma management and prevention Study. A total of 592 subjects were recruited from the Asthma Genome Research Center comprising hospitals of Soonchunhyang Seoul and Bucheon Hospital, Chungnam, Chungbuk, and Seoul national university in Korea. All subjects provided informed consent, and the protocols were approved by the Institutional Review Board of each hospital. Each patient showed airway reversibility such as the inhalant bronchodilator-induced improvement with over 15% of forced expiratory volume in 1 s (FEV_1_) and/or the airway hyperresponsiveness to the provocative concentration of P20 methacholine [16]. Aspirin challenge was performed as previously described [17]. Subjects showing a fall rate of FEV_1 _less than 15% without skin manifestations were included in ATA group. The clinical profiles of subjects are listed in Table 1.

**Table 1:** Clinical profiles of AIA patients and ATA cases.

Clinical profile	Asthmatics (all subject)	AIA	ATA
N	592	163	429
Age of first medical examination (mean (range))	46.15 (15.40-77.88)	43.13 (17.22-72.73)	47.30 (15.40-77.88)
Height (cm)	160.78 ± 8.63	161.72 ± 8.69	160.42 ± 8.39
Weight (kg)	62.81 ± 10.84	61.25 ± 10.38	63.40 ± 10.97
Fall rate (%)	9.27 ± 13.24	24.63 ± 16.11	3.54 ± 4.85
Blood eosinophil (%)	6.01 ± 5.73	5.96 ± 5.21	6.03 ± 5.92
FVC %, predicted	88.54 ± 14.08	90.35 ± 14.04	87.85 ± 14.05
FEV_1 _%, predicted	90.54 ± 16.97	87.58 ± 16.94	91.66 ± 16.87
PC20, methacholine (mg/ml)	6.43 ± 8.67	5.02 ± 7.83	6.91 ± 8.90
Total IgE (IU/ml)*	156 (62, 394)	164 (78, 357)	154 (53, 416)
Sex (male/female)	206/386	59/104	147/282
Current Smoker (%)	27.70	21.47	30.07
Positive rate of skin test (%)	56.42	52.76	57.81

Fall rate refers to the decline of FEV_1_% by aspirin provocation.

*Total IgE value is shown as median (25% interquartile interval, 75% interquartile interval).

AIA, aspirin-intolerant asthma; ATA, aspirin-tolerant asthma; FVC, forced vital capacity; FEV_1_, forced expiratory volume in 1 second.

All patients had a history of dyspnea and wheezing during the previous 12 months, plus one of the following: 1) >15% increase in FEV1 or >12% increase plus 200 mL following inhalation of a short-acting bronchodilator, 2) <10 mg/mL PC20 methacholine, and 3) >20% increase in FEV1 following 2 weeks of treatment with inhaled steroids and long-acting bronchodilators. Twenty-four common inhalant allergens were used for a skin prick test [9]. Total IgE was measured by the CAP system (Pharmacia Diagnostics, Uppsala, Sweden). Atopy was defined as having a wheal reaction equal to or greater than histamine or 3 mm in diameter. The asthmatic patients had experienced no exacerbation of asthma and respiratory tract infection in the 6 weeks preceding oral aspirin challenge (OAC). OAC was performed with increasing doses of aspirin using methods slightly modified from those described previously [9,18]. Changes in FEV_1 _were followed for 5 hours after the last aspirin challenge dose. Aspirin-induced bronchospasms, as reflected by rate (%) of FEV_1 _decline, were calculated as the pre-challenge FEV_1 _minus the post-challenge FEV_1 _divided by the pre-challenge FEV_1_. OAC reactions were categorized into 2 groups as follows: 15% or greater decreases in FEV_1 _with naso-ocular or cutaneous reactions (aspirin intolerant asthma: AIA), and less than 15% decreases in FEV_1 _without naso-ocular or cutaneous reactions (aspirin tolerant asthma: ATA).

### SNP selection and genotyping

We selected candidate SNPs showing polymorphic in the National Center for Biotechnology Information (build 36), and then genotyped in 163 AIA and 429 ATA subjects. Genotyping was performed at a multiplex level of using the Illumina Golden Gate genotyping system [19] and data quality was assessed by duplicate DNAs (n = 10). The genotype quality score for retaining data was set to 0.25. SNPs that could not satisfy the following criteria were excluded: (i) a minimum call rate of 90%; (ii) no duplicate error; (iii) Hardy-Weinberg equilibrium greater than *P *> 0.001. A total of 66 SNPs from *FSIP1 *were successfully genotyped.

### Statistics

We examined Lewontin's D' (|*D'*|) and the linkage disequilibrium (LD) coefficient *r*^2 ^between all pairs of biallelic loci. Linkage disequilibrium was inferred using the algorithm developed by the Broad Institute (using the program Haploview) [20]. Haplotypes were first estimated by using PHASE software [21], and then computed by logistic analyses using the Statistical Analysis System (SAS). Subjects harboring missing genotypes were omitted in the analysis of individual single-nucleotide polymorphisms and haplotypes. The genotyping success rate was >99%, so it is unlikely that omitting a small number of individuals introduced any bias in the analysis. The genotype and haplotype association with AIA were analyzed using logistic models with age (continuous value), gender (male = 0, female = 1), smoking status (non-smoker = 0, ex-smoker = 1, smoker = 2), atopy (absence = 0, presence = 1), and BMI as covariates. Significant associations are shown in bold face (*P *< 0.05). The common alleles were used as the referent genotype to the heterozygote and homozygote of the minor allele in referent analysis. The association analyses of differences in the fall rates in FEV_1 _following aspirin challenge with the genotypes and haplotypes were examined by regression analysis using SAS. The data were managed and analyzed using SAS. The effective numbers of independent marker loci in each genes were calculated to correct for multiple testing using the software SNPSpD [22], which is based on the spectral decomposition (SpD) of matrices of pair-wise LD between SNPs [22].

## Results

### Subject Characteristics

A total of 592 asthma patients were recruited for this study, and there were 163 AIA patients and 429 ATA cases (Table 1). First of all, the fall rate by aspirin provocation in AIA patients showed significantly higher fall rate (24.63%) than ATA cases (3.54%) (*P *< 0.0001, Table 1). Among the total subjects, 27.70% were current smokers, with more smokers within ATA patients (30.07%) than AIA patients (21.47%). Also, the positive skin test rate of AIA was smaller than that of ATA patients (52.76 and 57.81, respectively).

### SNP Analyses

**Figure 1:**
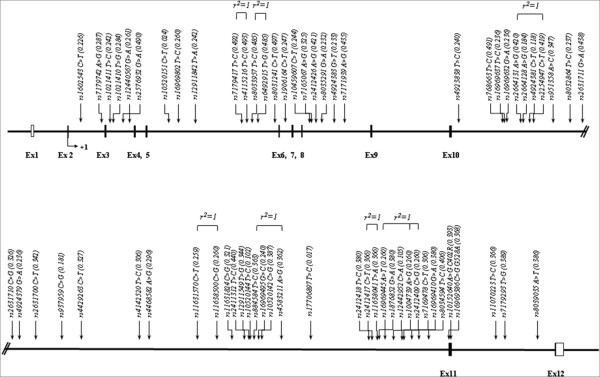
**A physical map of *FSIP1 *on chromosome 15q14**. Exons, introns, UTR, and SNPs are shown. Coding exons are marked by shaded blocks; UTRs by white blocks. SNPs with complete LD are represented by *r*^2 ^= 1.

Figure 1 shows the physical map of *FSIP1*, which contains 66 SNPs of the *FSIP1 *gene along with positions at exons and introns. Most of the variants were located in intronic regions (Additional File 1). In case of SNPs in coding region, two nonsynonymous SNPs, *rs10152640 *and *rs16969386*, were located in exon11 region, and they also caused amino acid change, with the former causing cysteine to arginine amino acid change at position 402, while the latter caused glycine to alanine amino acid change at position 528.

In logistic analysis for associations between SNPs of *FSIP1 *and AIA in a Korean population with adjustment for age, sex, smoking status, atopy and BMI, there was 18 SNPs showing initial significant associations and one SNP showing significant association after correction (*rs7179742*, *P *= 0.0007, *P*^*cor*. ^= 0.03, OR = 1.63, 95% CI = 1.23-2.16). Detailed analyses of *rs7179742*, along with other SNPs in the same haplotype block (*FSIP1_BL1*), are shown in Table 2. Although *rs7179742 *was the only SNP with significant relation after correction for multiple testing, SNPs in *FSIP1_BL1 *had considerably significant *P*-values before correction for multiple testing. Furthermore, SNPs in *FSIP1_BL1 *including *rs7179742 *were observed to have an effect on the increased fall rate of FEV_1 _by aspirin provocation (Table 3).

**Table 2:** Logistic and statistical analyses of *FSIP1 *SNPs in haplotype block 1.

rs#	Position	MAF			Heterozygosity	HWE	OR(95%CI)	*P*	*P*^*cor*.^
							
		AIA	ATA	Total					
*rs1602543*	Intron2	0.199	0.235	0.226	0.350	0.997	0.81 (0.59-1.11)	0.19	1.00
*rs7179742*	Intron2	0.358	0.262	0.287	0.409	0.765	1.63 (1.23-2.16)	**0.0007**	**0.03**
*rs1021411*	Intron3	0.292	0.227	0.242	0.367	0.885	1.47 (1.09-1.98)	**0.01**	0.55
*rs1021410*	Intron3	0.352	0.262	0.284	0.407	0.751	1.60 (1.20-2.13)	**0.001**	0.06
*rs12440567*	Intron3	0.325	0.240	0.261	0.386	0.788	1.58 (1.18-2.10)	**0.002**	0.09
*rs2576932*	Intron3	0.443	0.508	0.490	0.500	0.786	0.75 (0.58-0.98)	**0.03**	1.00
*rs10520151*	Intron5	0.027	0.023	0.024	0.047	0.274	1.17 (0.53-2.59)	0.70	1.00
*rs16969802*	Intron5	0.322	0.239	0.260	0.384	0.705	1.56 (1.17-2.08)	**0.002**	0.11
*rs12911842*	Intron5	0.211	0.260	0.242	0.367	0.467	0.73 (0.54-0.99)	**0.04**	1.00
*rs7179417*	Intron5	0.446	0.509	0.492	0.500	0.815	0.76 (0.59-0.98)	**0.04**	1.00
*rs4115216*	Intron5	0.446	0.510	0.493	0.500	0.877	0.76 (0.58-0.98)	**0.03**	1.00
*rs8033957*	Intron5	0.527	0.468	0.485	0.500	0.511	1.28 (1.00-1.66)	**0.05**	1.00
*rs6492915*	Intron5	0.527	0.468	0.485	0.500	0.511	1.28 (1.00-1.66)	**0.05**	1.00
*rs8031241*	Intron5	0.452	0.513	0.497	0.500	0.905	0.76 (0.59-0.99)	**0.04**	1.00
*rs1906164*	Intron6	0.232	0.249	0.247	0.372	0.852	0.91 (0.67-1.23)	0.53	1.00

MAF, minor allele frequency; AIA, aspirin-intolerant asthma; ATA, aspirin-tolerant asthma; HWE, Hardy-Weinberg Equilibrium;

OR, odds ratio; CI, confidence interval; *P*^*cor*.^, corrected *P*-value.

**Table 3:** Association analysis between SNPs of *FSIP1 *gene and the fall of FEV_1 _by aspirin provocation.

rs#	C/C		C/R		R/R		P*
				
	n	Fall rate	n	Fall rate	n	Fall rate	
*rs1602543*	356	9.74 ± 13.12	205	8.25 ± 13.03	31	9.88 ± 15.55	0.43
*rs7179742*	300	8.29 ± 12.05	241	9.49 ± 14.15	51	13.54 ± 14.55	**0.01**
*rs1021411*	335	8.84 ± 12.89	223	9.58 ± 13.87	34	10.88 ± 12.14	0.26
*rs1021410*	299	8.30 ± 12.03	245	9.56 ± 14.13	48	13.38 ± 14.86	**0.01**
*rs12440567*	322	8.36 ± 12.33	227	9.81 ± 14.15	43	12.76 ± 14.11	**0.02**
*rs2576932*	157	11.33 ± 15.8	290	8.34 ± 11.80	144	8.76 ± 12.69	0.06
*rs10520151*	564	9.15 ± 13.02	27	0.71 ± 17.32	1	13.8	0.50
*rs16969802*	324	8.38 ± 12.32	225	9.80 ± 14.18	43	12.68 ± 14.14	**0.02**
*rs12911842*	341	9.57 ± 13.93	210	9.30 ± 12.54	41	6.09 ± 9.94	0.14
*rs7179417*	156	11.40 ± 15.8	291	8.25 ± 11.78	145	8.87 ± 12.64	0.06
*rs4115216*	155	11.46 ± 15.8	292	8.23 ± 11.77	145	8.87 ± 12.64	0.06
*rs8033957*	162	9.08 ± 13.21	284	8.22 ± 11.59	145	11.41 ± 15.8	0.10
*rs6492915*	162	9.08 ± 13.21	284	8.22 ± 11.59	145	11.41 ± 15.8	0.10
*rs8031241*	153	11.52 ± 15.9	291	8.20 ± 11.76	147	8.91 ± 12.62	0.06
*rs1906164*	339	9.25 ± 12.60	216	9.21 ± 13.94	37	9.24 ± 14.74	0.98

*P-values of regression analysis represent the co-dominant model controlling sex, smoking status, and atopy as covariates.

C/C, C/R and R/R indicate the homozygote of common allele, and the heterozygote and homozygote of rare allele, respectively.

Fall rate values are mean ± standard deviation.

**Figure 2:**
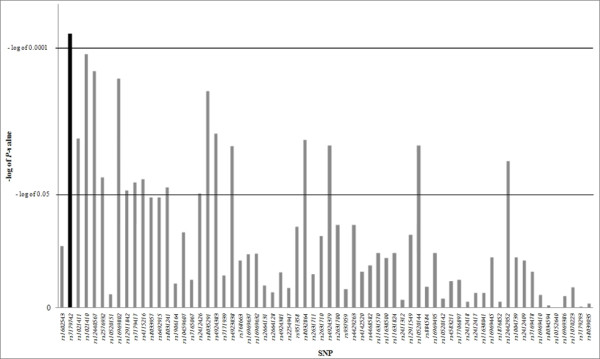
**A graph of negative log of P-values of SNPs**. After correction for multiple testing, all SNPs are calculated for the better representation of the values. The log = 0.0001 equals to *P *= 0.05. SNP *rs7179742*, which is the only SNP to have significant *P*-value after the multiple corrections, is highlighted in black.

Logarithms for *P*-values of all SNPs were calculated for the better representation of the values (Figure 2). Simultaneously, negative log of 0.05 and 0.0001, where latter value equals to 0.05 after correction for multiple testing (multiple testing correction number = 49.39), show the lines for significant association. In particular, SNPs with its value exceeding lower line (negative log of 0.05) would have initial association with AIA, and if it exceeds upper line (negative log of 0.0001), it would still have significant association after correction for multiple testing. As shown in Figure 2, there were 18 SNPs out of 66 that showed initial significant association with AIA. 12 SNPs out of the 18 showed association with the susceptibility to the risk of AIA, furthermore, rs7179742 had significantly higher MAF for AIA than that of ATA, showing increased susceptibility of AIA (MAF = 0.358 and 0.262, respectively). On the other hand, six SNPs out of the 18 showed lower minor allele frequencies in AIA patients compared to those of ATA cases.

### Haplotypes Analyses

**Figure 3:**
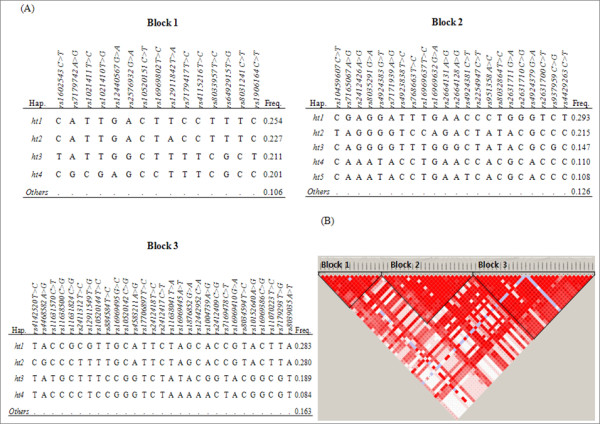
**Haplotypes and LDs of *FSIP1 *gene**. (A) Haplotypes of *FSIP1 *in the Korean population. They are divided into 3 haplotype blocks. Only those with frequencies over 0.05 are shown. (B) LD coefficients graphical plot (|D'|) among SNPs based on the genotypes of whole study subjects in this study (n = 592).

Haplotypes were calculated using PHASE software, and there were 3 haplotype blocks identified (Figure 3). Interestingly, pair-wise comparisons showed tight LD with three LD blocks among 66 SNPs of the *FSIP1 *gene. From each haplotype blocks, a total of 13 haplotypes with frequencies higher than 0.05 are listed (Figure 3A). Among them, 4 haplotypes showed initial association with AIA, as detailed analyses of the haplotypes are listed in Table 4. Three haplotypes, *FSIP1_BL1_ht4 *(*P *= 0.03), *FSIP1_BL2_ht4 *(*P *= 0.02), *FSIP1_BL3_ht3 *(*P *= 0.04) and *FSIP1_BL3_ht4 *(*P *= 0.01), were frequent in AIA patients, whereas *FSIP1_BL3_ht3 *revealed a lower frequency in AIA compared to that of ATA cases, respectively. However, their signals disappeared after the correction for multiple testing.

**Table 4:** Logistic and statistical analyses of *FSIP1 *haplotypes.

Haplotypes	MAF			Heterozygosity	HWE	OR (95%CI)	*P*	*P*^*cor*.^
						
	AIA	ATA	Total					
*FSIP1-_ BL1_ht1*	0.235	0.256	0.254	0.379	0.770	0.91 (0.67-1.23)	0.52	1.00
*FSIP1-_BL1_ ht2*	0.202	0.241	0.227	0.351	0.683	0.76 (0.55-1.03)	0.08	1.00
*FSIP1-_BL1_ ht3*	0.187	0.214	0.208	0.330	0.498	0.85 (0.62-1.16)	0.30	1.00
*FSIP1-_BL1_ ht4*	0.244	0.190	0.202	0.322	0.546	1.42 (1.04-1.94)	**0.03**	1.00
*FSIP1-_BL2_ ht1*	0.262	0.302	0.295	0.416	0.614	0.82 (0.62-1.09)	0.18	1.00
*FSIP1-_BL2_ ht2*	0.199	0.226	0.214	0.336	0.207	0.82 (0.60-1.11)	0.20	1.00
*FSIP1-_BL2_ ht3*	0.130	0.148	0.146	0.250	0.400	0.88 (0.61-1.27)	0.49	1.00
*FSIP1-_BL2_ ht4*	0.151	0.098	0.111	0.197	0.908	1.61 (1.10-2.37)	**0.02**	0.76
*FSIP1-_BL2_ ht5*	0.123	0.104	0.109	0.195	0.839	1.34 (0.89-2.01)	0.17	1.00
*FSIP1-_BL3_ ht1*	0.238	0.291	0.283	0.406	0.011	0.78 (0.59-1.04)	0.09	1.00
*FSIP1-_BL3_ ht2*	0.298	0.269	0.278	0.401	0.600	1.21 (0.91-1.60)	0.19	1.00
*FSIP1-_BL3_ ht3*	0.157	0.202	0.187	0.304	0.575	0.70 (0.50-0.98)	**0.04**	1.00
*FSIP1-_BL3_ ht4*	0.117	0.072	0.085	0.155	0.200	1.76 (1.13-2.74)	**0.01**	0.66

MAF, minor allele frequency; AIA, aspirin intolerant asthma; ATA, aspirin tolerant asthma; HWE, Hardy-Weinberg equilibrium number; OR, odd ratio; CI, confidence interval; *P*^*cor*.^, corrected *P*-value.

## Discussion

Most of the SNPs were located in intronic region, but two SNPs, *rs10152640 *and *rs16969386*, were located in exon region. They also caused amino acid change, as *rs10152640 *caused cysteine to arginine amino acid change at position 402 and *rs16969386 *caused glycine to alanine amino acid change at position 528. Nonsynonymous amino acid changes have significant effect on the expression and function of the protein [23,24]. In particular, the hydrophobicity change from cysteine to arginine (2.5 and -4.5 of hydrophobicity, respectively) on the *rs10152640 *could induce a critical conformational or functional change of the protein. However, when the association between two nonsynonymous SNPs of *FSIP1 *and the fall rate of FEV_1 _by aspirin provocation was analyzed, no significant association was found (data not shown), indicating that these nonsynonymous variants could not have a direct effect on the activities of FSIP1 protein.

From the association analyses of *FSIP1 *gene with AIA, we initially found 18 polymorphisms that showed association. In addition, significant associations of several SNPs in *FSIP1_BL1 *and *FSIP1_BL2 *with the fall rate of FEV_1 _by aspirin provocation were found (data for *FSIP1_BL2 *not shown). However, after multiple testing correction, only *rs7179742 *(*P *= 0.0007, *P*^*cor*. ^= 0.03, OR = 1.63, 95% CI = 1.23-2.16) showed increased susceptibility of AIA. The possible susceptibility of *rs7179742 *to AIA was reinforced by the significant association of this variant with the increased fall rate of FEV_1 _by aspirin provocation (*P *= 0.01 in co-dominant model, Table 3). Although this SNP is located in the intronic region of the gene, intronic region can still affect overall transcription in different ways. It was reported that introns can affect transcription efficiency [25], and another study demonstrated that variations in intronic region can lead to splicing abnormalities, which in turn may lead to human diseases [26]. It was also reported recently that a SNP located in intronic region showed an association with asthma [27]. On the other hand, in additional analysis of LD near *FSIP1 *in Asian populations (Chinese and Japanese) from the International HapMap Project http://hapmap.ncbi.nlm.nih.gov/, the *FSIP1 *gene is in LD with the *thrombospondin-1 *(*THBS1 *or *TSP-1*) gene (Additional File 2). The *THBS1 *gene has been implicated in the network underlying the pulmonary response to oxidative stress in asthma [28]. More interestingly, aspirin, as an inhibitor of THBS1, has recently shown to lead to reduction in THBS1 levels [29]. This suggests that *FSIP1 *might have an effect on aspirin hypersensitivity in asthma, with relation to the nearby potential gene of *THBS1*.

The *FSIP1 *gene has been discovered very recently. *FSIP1 *is regulated by APP, an integral member protein that is found in many tissues. APP, in turn, is cleaved by an asthma susceptible gene called *ADAM33 *[30]. *ADAM33 *polymorphisms have been associated with asthma risk in various populations, including Korea [31], Thai, Han Chinese and Japanese [32-34], who are closely related with Korean population, especially Han Chinese and Japanese. The *ADAM33 *polymorphisms have also been related with asthma risk of smoking population [35,36]. These results suggest that the *FSIP1 *gene could have a correlation with APP and/or *ADAM33 *in development of asthma. In addition, the fact that aspirin affects wide range of human body, from lung to blood clotting to spermatogenesis, has to be considered. Naturally, the mechanism and pathways of aspirin is intricate and complicated.

One of limitation of our study is the small number of AIA patient, which might result in decreasing the statistical power of this study. However, considering the rareness of AIA condition, our results could provide supporting information, with further needs of replication in large number of subjects. Taken together, even though current knowledge of *FSIP1 *gene implicates that it only affects a small part out of aspirin pathways, our findings suggest a new relation between *FSIP1 *and aspirin hypersensitivity that explains the association found in this study.

## Conclusions

In summary, we found 18 SNPs and 4 haplotypes of *FSIP1 *showing associations with AIA initially, and after multiple testing correction, one SNP, *rs7179742 *showed significant association with AIA. Although the relation between the *FSIP1 *gene and AIA is not yet clearly understood, our findings suggest that *FSIP1*-related regulations of APP and *ADAM33 *may play a role for the development of aspirin hypersensitivity in asthmatics, along with the fact that FSIP1 is expressed in airway epithelium. Future researches should concentrate on this part to discover more about the role of *FSIP1 *gene and also the relation between the gene and various diseases including AIA.

## Abbreviations

*FSIP1*: *Fibrous sheath-interacting protein 1*; AIA: Aspirin-intolerant asthma; ATA: Aspirin-tolerant asthma; APP: Amyloid beta (Aβ) precursor protein; *ADAM33*: *α disintegrin and metalloproteinase 33*; SNP: Single nucleotide polymorphism; BMI: Body mass index; FEV_1_: Forced expiratory volume in 1 second; IgE: Immuglobulin E; OAC: Oral aspirin challenge; PAF: Platelet activating factor; ht: Haplotype; FVC: Forced Vital Capacity; *P*: *P *value, and *P*^*cor*.^: Corrected *P *value; LD: Linkage Disequilibrium; THBS1: Thrombospondin-1

## Competing interests

The authors declare that they have no competing interests.

## Authors' contributions

JYK and JHK developed tables/figures, and drafted the manuscript. TJP, JSL, and CFP participated in preparation and quality control of samples. JSB, BLP, and HSC performed the statistical analysis. JSP, SWP, STU, MKK, ISC, SHC, and BWC helped to recruit subjects. CSP and HDS managed all of this study and helped to draft the manuscript. All authors read and approved the final manuscript.

## Pre-publication history

The pre-publication history for this paper can be accessed here:

http://www.biomedcentral.com/1471-2466/10/34/prepub

## Supplementary Material

Additional file 1 **Supplementary Table 1- SNPs of *FSIP1 *and their *P*-value.** The table contains all the SNPs of FSIP1 examined in our study and their P-value. This includes SNPs from haplotype block 2 and 3. Click here for file

Additional file 2 **Supplementary Figure 1 - LD plot nearby *FSIP1***. The LD near *FSIP1 *in Asian populations (Chinese and Japanese) is analyzed from the International HapMap Project http://hapmap.ncbi.nlm.nih.gov/. LD coefficient (D') among SNPs of *THBS1*, *FSIP1*, and *GPR176 *in Asian populations. The *FSIP1 *is in LD with *THBS1 *with a LD block. Click here for file
