# Supplementary material for: Positive Association between Aspirin-Intolerant Asthma and Genetic Polymorphisms of FSIP1: a Case-Case Study
Source: BMC Pulm Med. 2010 Jun 1;10:34. doi: 10.1186/1471-2466-10-34 (PMC2896935; doi:10.1186/1471-2466-10-34)
Supplement: Additional file 1 — Supplementary Table 1- SNPs of FSIP1 and their P-value. The table contains all the SNPs of FSIP1 examined in our study and their P-value. This includes SNPs from haplotype block 2 and 3. [file 1471-2466-10-34-S1.DOCX]

Supplementary Table 1. SNPs of *FSIP1* and their *P*-value.

| rs# | *P*-value | rs# | *P*-value | rs# | *P*-value |
| --- | --- | --- | --- | --- | --- |
|  |  |  |  |  |  |
| *rs1602543* | 0.19 | *rs768663* | 0.28 | *rs10520144* | **0.01** |
| *rs7179742* | **0.0007** | *rs16969637* | 0.24 | *rs884584* | 0.57 |
| *rs1021411* | **0.01** | *rs16969632* | 0.24 | *rs16969495* | 0.23 |
| *rs1021410* | **0.001** | *rs2664131* | 0.55 | *rs10520142* | 0.78 |
| *rs12440567* | **0.002** | *rs2664128* | 0.66 | *rs4583211* | 0.49 |
| *rs2576932* | **0.03** | *rs4924381* | 0.39 | *rs17706897* | 0.48 |
| *rs10520151* | 0.70 | *rs2254947* | 0.59 | *rs2412418* | 0.84 |
| *rs16969802* | **0.002** | *rs951358* | 0.12 | *rs2412417* | 0.67 |
| *rs12911842* | **0.04** | *rs8032864* | **0.01** | *rs11638041* | 0.68 |
| *rs7179417* | **0.04** | *rs2631711* | 0.41 | *rs16969445* | 0.26 |
| *rs4115216* | **0.03** | *rs2631710* | 0.15 | *rs1876852* | 0.84 |
| *rs8033957* | **0.05** | *rs4924379* | **0.01** | *rs12442952* | **0.02** |
| *rs6492915* | **0.05** | *rs2631700* | 0.11 | *rs1004739* | 0.26 |
| *rs8031241* | **0.04** | *rs937959* | 0.61 | *rs2412409* | 0.28 |
| *rs1906164* | 0.53 | *rs4429263* | 0.11 | *rs7169478* | 0.38 |
| *rs10459607* | 0.14 | *rs4142520* | 0.38 | *rs16969410* | 0.71 |
| *rs7165067* | 0.47 | *rs4468582* | 0.32 | *rs8034594* | 0.94 |
| *rs2412426* | **0.05** | *rs11631570* | 0.23 | *rs10152640* | 0.99 |
| *rs8035291* | **0.003** | *rs11638500* | 0.27 | *rs16969386* | 0.73 |
| *rs4924383* | **0.01** | *rs11631824* | 0.23 | *rs11070223* | 0.58 |
| *rs7171939* | 0.42 | *rs2411312* | 0.81 | *rs7179293* | 0.97 |
| *rs4923838* | **0.01** | *rs12911549* | 0.14 | *rs8039035* | 0.90 |

*P*-values were calculated using SAS.
