# Supplementary figures and images for: Positive Association between Aspirin-Intolerant Asthma and Genetic Polymorphisms of FSIP1: a Case-Case Study
Source: BMC Pulm Med. 2010 Jun 1;10:34. doi: 10.1186/1471-2466-10-34 (PMC2896935; doi:10.1186/1471-2466-10-34)

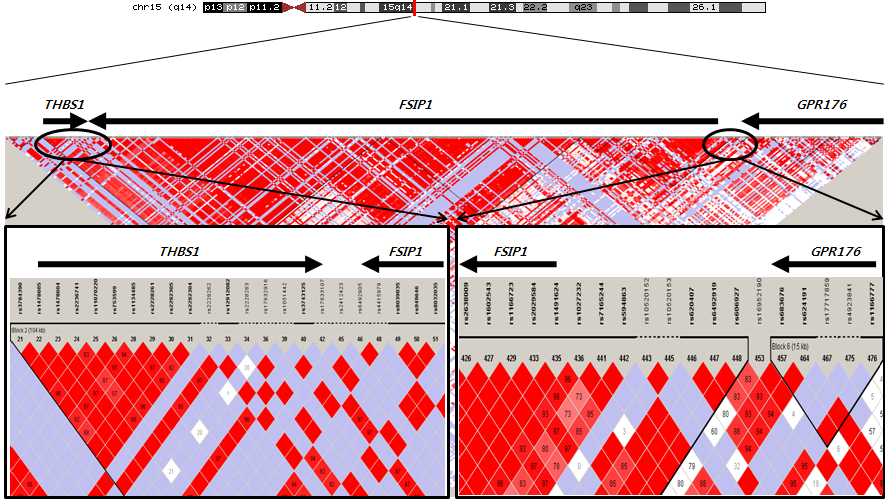

Supplement: Additional file 2 — Supplementary Figure 1 - LD plot nearby FSIP1. The LD near FSIP1 in Asian populations (Chinese and Japanese) is analyzed from the International HapMap Project http://hapmap.ncbi.nlm.nih.gov/. LD coefficient (D') among SNPs of THBS1, FSIP1, and GPR176 in Asian populations. The FSIP1 is in LD with THBS1 with a LD block. [file 1471-2466-10-34-S2.TIFF]
